# An Introduction to Engaged Phenomenology

**DOI:** 10.1080/00071773.2022.2081533

**Published:** 2022-06-29

**Authors:** Jessica Stanier

**Affiliations:** Wellcome Centre for Cultures and Environments of Health, Politics Department, University of Exeter, Exeter, UK

## Abstract

In this article, I introduce engaged phenomenology as an approach through which phenomenologists can more explicitly and critically consider the generative conditions and implications of their research. I make an explicit link between philosophical insights from critical and generative phenomenology and the ethical and methodological insights offered by engaged research methods—a community-oriented approach to the generation of shared understanding for the mutual benefit of all stakeholders in research. The article consists of (a) a review of these respective strands of inquiry, (b) an overview and critique of mainstream qualitative methodologies in phenomenology, and (c) suggestions for those interested in working through engaged phenomenology as an approach to both theory and research praxis.

In attending to phenomena as they are lived through in conscious experience, phenomenology is sometimes accused of naively eschewing analysis of the social, political, and historical structures which imbue experiences with shared meaning.^1^ The phenomenological method can give the impression of an almost Cartesian project, bent on reconstructing philosophical grounds for reality from the perspective of a sole, independent, and autonomous subject. The apparent hubris of such an undertaking—not to mention its concomitant methodological limitations, occlusions, and foreclosures—has been strongly critiqued by opponents and proponents of the phenomenological approach alike.^2^ In particular, phenomenologists have been accused of taking experience as a sole foundation for knowledge, framing experience problematically as ahistorical, assuming that experience is immediately accessible for analysis, and failing to take into account the interpretative dimension of experiential constitution.^3^

To most working in the discipline today, however, it is clear that these critiques have not proved fatal to the phenomenological project, but have instead enlivened and enriched debate along these lines.^4^ Though there is a long history of phenomenologists linking their work to other disciplines and to social and political issues, those who have perhaps most robustly responded to these critiques have tended to thematize the practice of phenomenology as *participation* in the very worldly phenomena it purports to describe.^5^ The idea that the phenomenologist effects change in the world through phenomenological reflection—either inadvertently or deliberately, passively or actively—is as radically promising as ever.^6^ Shifting away from an uncritical notion of the “now,” contemporary phenomenological approaches are increasingly engaging with their situated cultural and historical contexts, both through critical reflection and through interdisciplinary research collaborations.^7^ These robust new theoretical avenues importantly integrate the contingency of the phenomenological approach, its very concepts, and its participation in intersubjective meaning-complexes into philosophical understanding.

These conceptions of phenomenology as *activity* have been recently underscored by pandemic-related disruption, during which the phenomenological method has been experienced as highly contingent on structural conditions of possibility. This disruption has revealed the extent to which it is easy for phenomenologists to take for granted the structures which ordinarily enable research activity to proceed according to academic norms and conventions; indeed, some have reported returning to their phenomenological studies “changed, stretched, [and] transformed” by these experiences.^8^ These timely observations affirm how sense-making—both as active reflection and as passive constitution—is animated by specific historical and relational contexts, which are always incorporated into the foreground and background of experience as lived through. Crucially, we are reminded that phenomenology itself is a temporal process which happens *somewhere* and *somewhen* (for *someone*). Everyone, including phenomenologists, are situated within particular contexts, and this inevitably affects how they make sense of experience or conduct their research. Indeed, very particular constellations of conditions and structures are necessary to enable and sustain academic studies of this kind.

While new approaches are beginning to take these structures into account in a *theoretical* sense, it is my contention that phenomenologists could carry these reflections to more radically engaged conclusions. In this regard, however, there is no need to reinvent the wheel. Drawing from engaged research literature—an approach which is already extensively established—I propose the notion of *engaged phenomenology* as an invitation for phenomenologists to more explicitly and critically consider the generative conditions and implications of their research. Engaged research, as I discuss in more detail later in this introductory article, is a community-oriented approach to the generation of shared understanding for the mutual benefit of all stakeholders. This connection with engaged research matters in more ways than one. There has been a proliferation of “named” approaches to phenomenology in recent years—including critical, applied, generative, neurophenomenological, and micro-phenomenological variants—and it is not my intention to needlessly muddy the waters with another neologism.^9^ Indeed, the engaged approach is, in principle, applicable to any of these variants of phenomenology; one could undertake an engaged micro-phenomenological study or critical-phenomenological project, for example. As I explore in this introduction to engaged phenomenology, the name is intended to citationally orient those who may be interested in taking up this invitation on ethical, epistemological, and methodological levels. As Ahmed instructively points out, “[c]itation is how we acknowledge our debt to those who came before; those who helped us find our way when the way was obscured because we deviated from the paths we were told to follow”.^10^ As self-styled “perpetual beginners,” I here invite phenomenologists to explore these alternative pathways and to realize the generative potential of engaged research as an approach.

## 1. Critical Generativity in Phenomenological Research

At least two branches of contemporary phenomenology have already offered important attempts to more explicitly thematize the project of *research* in the manner discussed above: to foreground the socio-historical specificity of researchers’ interests and commitments, and to value the transformative nature of research itself without recourse to the illusion of a neutral “now-point” from “no-where”.^11^ Critical phenomenology is one of these branches. Critical phenomenologists respond to the fact that, in practising phenomenology, one already cares “about that within which one appears as phenomenon”—and this care is shaped by social and political structures pertaining to a situated context which one might seek to change.^12^ In particular, the project arises out of the intersectional concerns of gender, critical race, queer, and disability scholarship and activism,^13^ among others, where the combination of lived experience and theory has long been identified as a means of “collective liberation”.^14^ In this context, the phenomenological toolkit has been inherited, appropriated, subverted, and otherwise fruitfully applied.^15^ As Guenther summarizes, critical phenomenology seeks to expose and analyse the “norms of our lifeworld” which constitute “how we make sense of things” in order to effect “liberation from the structures that privilege, naturalize, and normalize certain experiences of the world while marginalizing, pathologizing and discrediting others”.^16^ Indeed, Marx’s famous formulation in the *Theses on Feuerbach*—“philosophers have only interpreted the world, in various ways; the point is to change it”—is paralleled by Weiss, Murphy, and Salamon in their definition of the critical-phenomenological enterprise as “an ameliorative phenomenology that seeks not only to describe but also to repair the world”.^17^

The second branch worth mentioning here, as having constructively thematized research itself within the phenomenological endeavour, is that of generative phenomenology. This approach more closely stems from the traditional phenomenological canon, though some critical phenomenologists have also associated themselves with this branch.^18^ Generative phenomenology, as Oksala puts it, “questions the traditional phenomenological assumption that sense-constitution begins with an individual subject rather than extending beyond him or her and stemming from tradition, culture, language and history”.^19^ By incorporating these cultural and historical dimensions into phenomenological analysis, generative phenomenology is able to thematize collective sense-making; in this way, it exceeds static and genetic phenomenologies which focus on individual streams of consciousness, and therefore lack the capacity to take on more critical and political valances.^20^ These generative phenomena of culture and history, among others, are never fully and directly “given to the individual subject in experience, nor can they ever concern only one person, yet they are constitutive features in world constitution”.^21^ In short, the generative phenomenologist is “not concerned merely with the structure of generation, but with how one generates structure”.^22^

In this way, generative phenomenological research is able to critically respond to its own conditions of possibility—since, as Steinbock notes, “the generative phenomenologist and phenomenology stand within a specific historicity,” and there is a “singularity or uniqueness” to any individual generative phenomenologist’s interests.^23^ Generative phenomenologists are therefore called upon to regard their work as *both* critical description *and* normative participation; the activity of “doing phenomenology” is conceived as temporally integrated and ethically connected to the objects of description. Indeed, these considerations are of methodological importance on multiple levels, such that the “phenomenologist must continually account for the changes that he or she introduces into generativity”.^24^

Approaches offered by critical and generative phenomenology thus allow the act of “doing phenomenology” to be conceived not simply as neutral description of the lifeworld and its modes of presentation, but rather as *participation* in the “things themselves” and thus situated within a specific socio-historical lifeworld. The phenomenologist here precisely *does not* purport to successfully take up an ahistorical and disinterested position in order to practice an abstraction of the world.^25^ The phenomenologist interested in critical generativity instead understands that they respond to, employ, and redirect the generative meaning-complex within which they undertake their inquiry, and that their activities concretely effect change within the lifeworld that they inhabit. The very practice of undertaking a phenomenological inquiry, therefore, entails a transformation of relations; the phenomenologist renews their understanding of certain phenomena in the world—at a particular time and in a particular place—through the activity of critical reflection, and this reflection generates a new orientation and world-view with respect to the lifeworld. Crucially, I would add, this activity of critical reflection is precisely made possible by certain material conditions within the lifeworld, without which the practice of phenomenology would not be possible. These conditions of possibility must not be taken for granted, and indeed they require active attendance and maintenance. It seems to me that these two branches of contemporary phenomenology offer rich theoretical resources for taking these conditions of possibility into account in ways that can shed new light on the practice of phenomenological research.

There are, however, relatively few studies arising out of either critical or generative phenomenology to have combined these theoretical insights with methods that involve working together with research participants to *collectively* critique or generate meaning-complexes.^26^ By contrast, there have been many researchers working through phenomenology who integrate qualitative research into their work. Before elaborating on the potential for engaged phenomenology, it is therefore worth summarizing these efforts, as well as their contributions, motivations, and relevance for an engaged phenomenological approach.

## 2. Mainstream Qualitative Methodology in Phenomenology

Phenomenology is sometimes reductively understood exclusively as a solo endeavour—an armchair exercise in adopting the epoché and the reduction while turning one’s attention to “the things themselves”.^27^ On the contrary, however, as Gallagher notes, phenomenologists often explore the experiences of others since this may “may help avoid the presuppositions that phenomenology wants to avoid, since one’s own imaginative faculties are limited by various biases or lack of knowledge”.^28^ To this end, phenomenologists have traditionally sought to learn about the experiences of others through the *second-hand* use of case studies.^29^ However, while this certainly opens up the horizons of the phenomenological inquiry from a single researcher’s imagination, the re-use of case studies usually precludes the possibility of checking any phenomenological conclusions in dialogue with the people referenced therein. Phenomenologists therefore rely heavily on their own interpretation when incorporating case studies into their work—since the phenomenological “data” has been, in these cases, elicited from participants by other researchers whose methods may be unclear, or, in other cases, the “data” originates from autobiographical anecdotes. Without critical reflection on the cultural discourses, narratives, and processes that constructed the “experience” as presented in a given case study, the phenomenologist risks problematically distorting other people’s experiences in order to evidence their own phenomenological claims.

Perhaps supplanting the use of case studies, there are increasing efforts to combine qualitative research with phenomenology^30^ and there are now numerous published accounts offering instructions on how to conduct a phenomenological interview firsthand.^31^ Phenomenological interview methods have been developed to enable researchers to more effectively “bracket” their own presuppositions about particular experiences, and to directly learn about these experiences in dialogue with participants. In contrast to case studies, interviews make it possible on a basic level for participants to speak for themselves. There is much debate over the extent to which these different integrations of phenomenology and qualitative research are successful, which I will not expound here.^32^ With a view to the potential of engaged phenomenology, however, I am especially concerned that some of these methods may inadvertently construct and overdetermine the very phenomenological descriptions or “data” that they seek, while seeming to sideline important ethical and epistemological considerations when working with participants.^33^

In their recent article “Critical phenomenology and psychiatry”, Zahavi and Loidolt claim that it is

“a distinctive strength of the phenomenological approach that it doesn’t merely speak out against scientific attempts to reify the other, but as a result of its commitment to respect and understand the subjective perspective of the other, also promotes an ethically responsive dialogue”.^34^

How exactly phenomenologists can concretely promote this “ethically responsive dialogue” remains an important methodological question, especially given this trend towards integrating qualitative research with phenomenology. There are consistent suggestions in phenomenological interview literature that “subjects” need to be better “skilled,” “trained,” “taught,” or “lead” through interviews.^35^ This is presented as a means to ensure that researchers are able to capture memories *as if* they were present experiences^36^ or *as if* they are “pristinely” free from normative construction.^37^ It is, however, impossible to access a participant’s experience directly as it is lived through, since it is always already recollected as memory retrospectively and narrated to a researcher in a very particular interpersonal context. At best, this aspect of recollection seems to be glossed over. At worst, these suggestions seem to demonstrate a fundamental misunderstanding about what goes on during an interview encounter. Krueger, Bernini, and Wilkinson are also critical of these claims, and argue incisively that this kind of approach “constructs the ‘pristine’ mental phenomena it purports to discover”, and that therefore:

when subjects are made to adopt an artificially passive and observational stance on their experiences, and then issue reports (guided by the interviewer), they transform what are initially world-directed vehicles dynamically lived through into objectified contents abstracted from the concrete relations and context that are part of their essential nature.^38^

Participants are always constructing a version of their experience for the interviewer, accommodating their expectations and following their direction.^39^ This is an inescapable phenomenological condition of recollection and narration—something which interviewers must accept and for which they must take ethical responsibility. By asking participants to strip down their experiences, and indeed by insisting that this is possible only if participants can learn to deliver correctly, interviewers risk enacting precisely the kind of ethically and epistemically problematic practice which, as we will see, is critiqued by the engaged research approach. Participants are asked to do the impossible—to offer narration through the mediation of the interview encounter, and yet without any narrative mediation at all—and therefore will inevitably “fall short”. When participants are asked to defer to interviewers in these ways, inappropriate power dynamics can foreclose opportunities to explore personal experiences that are important to participants and which they may wish to narrate differently; this much is widely acknowledged in social science literature but commentary on these matters is far less common in phenomenological interview methodologies.^40^ Not only does this run the risk that participants feel uncomfortable or unsafe in discussing sensitive experiences, but it also makes participation in interviews altogether inaccessible for many people who cannot “comply” with the demands of the interviewer. Many marginalized people can be thus excluded from research participation and academic knowledge creation.^41^ It is not that phenomenological interviews can never be useful—indubitably important research has arisen from these endeavours—but rather that claims over what these interviews are able to deliver are sometimes severely overstated and often, it seems, at cost to participants through their discomfort or exclusion. In this sense, these methods can precisely enact the “scientific attempts to reify the other” to which Zahavi and Loidolt claim phenomenology can offer an ethical alternative.^42^ So, while there is exciting promise in these attempts to integrate qualitative research and phenomenology, it is simply not sufficient to expect the phenomenological approach to deliver an ethical alternative without critical reflection on the broader context in which these interpersonal encounters are taking place.

It is my contention that, rather than being regarded as mere background to the phenomenological research process, the temporal dimensions and material conditions of the research process can be regarded as a generative locus of inquiry in multiple ways. Since researchers decide what aspects of complex phenomena matter most according to their normative research interests—to a greater or lesser extent, as influenced by the agendas of institutions and funders—this “introduces an unavoidable ethical component into our thinking” which must be addressed.^43^ This is where I argue that an engaged research approach, as theory and praxis, can offer a radical intervention and open up new horizons for phenomenological research.

## 3. Engaged Research

The term “engaged research” can refer to a range of practices, through which

[…] research is embedded in communities from the outset, not through “outreach” or “consultation” but through continuous co-creation, where the social goods of research in the form of remuneration, data, cultural capital and access to decision makers are generated in participation with communities and, ideally, equitably shared.^44^

It is an approach that has evolved out of participatory research, which first emerged from social movement and civil society structures in the global South and out of recognition for the importance and power of local post-colonial knowledge.^45^ Both engaged and participatory approaches question priorities assumed by researchers and highlight the power dynamics at play in research agendas and practices. Both, in principle, recognise that, by instigating a dialogue with participants, researchers often already determine the terms of address, as well as the norms associated with any expected response, in advance.^46^ Alongside this recognition of the interpellation involved in research, there is acknowledgement that researchers must nonetheless not turn away from the responsibility to produce, share, and act on situated knowledges to effect discursive and material change.^47^ Ideally, the impetus in these frameworks is on researchers to learn how to listen, rather than on participants to make themselves understood. Indeed, it is understood that researchers’ attempts to theorize others’ experiences *without* considered engagement can risk fundamentally misconstruing the meaning of a particular context, especially across significant power differentials.^48^ (Some theoretical acknowledgement of this can be found in critical-phenomenological literature).

Engaged research is an approach which has been especially taken up by researchers of health humanities and public participation, particularly with regard to cultural and political contexts of health and wellbeing.^49^ Differing from “public engagement”, which more often involves a focus on dissemination of research findings to the “general public”, engaged research entails working with communities on shaping research from its outset through a continuous or iterative process, such that the research can most equitably serve and openly respond to their situated priorities and needs.^50^ While engaged research is not synonymous with activism, it can provide an important basis for effecting change in communities both during and after the research process.^51^ This approach to research is also increasingly encouraged from an institutional perspective to evidence value, utility, and innovation to funders—a dynamic which itself can present its own ethical and practical challenges.^52^

The process of engagement with communities, who have lived experience that might inform research, necessitates a continual, evaluative, and self-reflexive dimension to projects. Engaged researchers necessarily come to understand their projects as enduring processes, made possible by dynamic networks of intersubjective relations and material infrastructures which culminate in “tide, flux and general unpredictability”.^53^ These projects often involve many people and “participation has to be continually re-negotiated”.^54^ By contrast, as discussed above, the conditions enabling *phenomenological* research as an activity can often recede into the background of the inquiry. It is nonetheless encouraging, as also discussed above, that critical and generative phenomenology already offer sophisticated means within the discipline with which to address these ethical and epistemological concerns as part of the process of phenomenological research. A cross-pollination with engaged research frameworks would, I contend, significantly help phenomenologists to better and more critically engage with the conditions out of which qualitative material has been rendered accessible to them, to “continually account for the changes that he or she introduces into generativity”,^55^ and to “not only […] describe but also […] repair the world”.^56^

## 4. Two Approaches to Engaged Phenomenology

To close this introduction to engaged phenomenology, I would like to here offer two concrete ways in which insights from engaged research might be put to work in the research agendas of phenomenologists today. Combined with the foregoing overview of relevant contemporary literature, it is my hope that these two approaches will help to orient those interested in taking up engaged phenomenology in their own work—or at least that this introduction will offer resources for critical self-reflection.

My first suggestion for those interested in taking up engaged phenomenology is to explicitly consider how a given phenomenological inquiry is situated within social, political, and institutional contexts that have made the research possible and which have framed its operative concepts and concerns. Not all phenomenologists are inclined to undertake or even collaborate on qualitative research, and, indeed, it is important that not everyone feels compelled to take this approach; most phenomenologists are, after all, trained in phenomenology first and foremost as a philosophical approach, and will quite understandably neither wish to nor feel prepared to depart from this kind of work. Nevertheless, this first approach to engaged phenomenology is just as applicable to more traditional phenomenologists as to more critically-minded researchers: “we all—no matter what our own expertise or topic of research—need to examine which sources, frameworks, and models we explicitly or implicitly foreground in the production, analysis, and dissemination” of research.^57^ This is already encouraged and well established among those working through critical phenomenology, as discussed above. Additionally, however, philosophical phenomenologists can pay careful attention to their reliance on case studies, and can critically reflect on “which disciplines, which theoretical perspectives, and which kinds of expertise have most authority in determining how concepts are defined”.^58^ This would involve reflecting on how operative concepts and key experiential structures have been foregrounded, and by whom, as well as questioning why methods or origins pertaining to a given experiential account remain tacit in a given source.

An engaged phenomenology of this sort would acknowledge explicitly that “[d]ifferent situations can alternately lead people to reveal or conceal their experiences, in turn altering what it is possible for others to recognise and receive”^59^ and would “remain attentive to how power structures the ways in which these experiences are rendered, legitimized, or ignored as ‘evidence’” in the context of research agendas.^60^ This approach would therefore also call for a consideration of how the phenomenological research itself, as an activity that affects change in the world, will influence discourses and serve particular interests (both within and beyond the academic sphere).^61^ Interdisciplinary phenomenological work that engages with cognitive science, psychiatry, and psychopathology, for example, could similarly engage with the fact that categories and diagnoses are *experienced* as interpellation; as Fernandez writes in his critical-phenomenological reflections on mad pride, “[i]f we are genuinely committed to identifying, assessing, and suspending our prejudices, then we ought to listen to those most affected by them”.^62^ Phenomenologists can in this way see themselves as “both influencing and influenced [by …] manifestations of cultural power,” can reflect on taken-for-granted parameters of their research, and can engage with their discursive effects more responsibly.^63^

The second approach to engaged phenomenology I suggest here is much more radical, in that it calls upon phenomenologists to engage far more directly and purposefully with the communities whose experiences are shaping the research. A project of this kind would require a significant degree of power-sharing with these communities, and would therefore call for a direct mobilisation of insights from the field of engaged research as described above. As Roth and Tobin describe, one of the most powerful outcomes of this kind of research is that “what has been learned is then available as a resource for action, hence agency, in the lifeworlds of the participants”.^64^ In other words, the fact that the research impacts the phenomena which it seeks to investigate is not simply an epistemological consideration; it actually becomes possible to design research that empowers those participating in the research through something like an “ethically responsive dialogue” as alluded to by Zahavi and Loidolt.^65^

This is not actually a proposal for an *entirely* new way of undertaking phenomenological research. In their own ways, many researchers working through phenomenology have already been adapting methodologies and methods in ways that speak to the practically engaged research approach. Vera-Gray, for instance, has developed her own phenomenologically-inspired interview approach as an alternative to frameworks in which the “structure and content remains defined by an outside source”.^66^ Recognizing that “for particular questions, settings and research relationships, conversation as method may gain the most robust data and generate the most useful knowledge,” Vera-Gray’s conversational approach takes the power dynamics of the interview situation into account and seeks to enable all participants to be involved in the “active construction of meaning”.^67^

Above and beyond a serious consideration of the ethical and epistemological implications of the interview encounter, however, this latter approach to engaged phenomenology would explore how best to meaningfully shape and share the research together with communities whose experiences are foregrounded in the study. The phenomenologically-aligned *Hearing the Voice* research project, for example, has taken stock of how working in this way with the voice-hearing community has afforded these people “rich possibilities […] in making sense of their experiences outside the relatively narrow frameworks of conventional psychiatric frameworks,” as well as exploring how “an interdisciplinary approach that foregrounds and values multiple forms of expertise—professional and experiential—can be fully integrated into mainstream […] research".^68^ By collaborating with participants—not only in the sharing of their experiences but also the *interpretation* of these experiences—it becomes possible to work together not only to critique and challenge but also to *generate* and co-create meaning-complexes.^69^ This approach therefore takes seriously the situatedness of phenomenological research as an intersubjective process, and has the capacity to explicitly address the fact that experiences are shaped by key concepts and power relations already defined in advance. There would be major scope for an engaged phenomenology of this sort to address what many, through Fricker, refer to as epistemic (hermeneutical) injustice,^70^ but that could be understood broadly through the impetus behind critical phenomenology to not only “describe but also to repair the world”.^71^

The challenges associated with an undertaking of this second kind should not be underestimated. Considerable investment is vital for resourcing and supporting the careful process of engagement, rapport-building, and meaningfully reciprocal relationships.^72^ Indeed, as researchers have considerable institutional influence and access to funds, this framework raises serious questions of ethical responsibility and accountability. Participation in engaged research projects is not always straightforwardly experienced as positively empowering, and can also be stressful, exhausting, and disappointing—particularly for participants whose time, health, and finances are more precarious, but also for researchers.^73^ Moreover, there will be no one single replicable framework or method that can instruct researchers how to sensitively undertake engaged phenomenology; as with any engaged research, this will always depend on the particular circumstances, needs, and priorities of a given community.^74^

To some extent, the contours of any overarching approach to engaged research will remain nebulous, and will necessitate attentive engagement with concrete relationships and conditions in practice. In the context of my own research, however, I ended up responding to this confluence of phenomenological currents—critical phenomenology, generative phenomenology, and engaged research—by writing something of a manifesto to describe how I envisaged engaged phenomenology in practice.^75^

*“Engaged phenomenology”, as an approach:*

  • *heeds the situatedness of lived experiences across diverse cultural and environmental lifeworlds;*
  • *invites us to hold this notion of plural lifeworlds together with wider phenomenological questions about lived possibility, power relations, and the condition of having and being in a lifeworld which feels open to us and to which we are open;*
  • *challenges assumptions around narrativity and privileged articulacy in phenomenological methods, embracing new ways of listening and attending to people’s lived experiences in their specificity and relationality;*
  • *is mindful of how experience is lived through constellations of relations with others, rather than only seeking individualised (depoliticised) first-hand accounts;*
  • *considers the transformative potential of research participants sharing their experiences in meaningful ways, rather than merely assessing their “utility” in academic terms.*

It is my sincere hope that this framework and overall introductory article will assist phenomenologists and practitioners in reflecting critically on their relational participation in the genesis of meaning for communities of many kinds—whatever path ultimately leads them towards the writing-table and its heretical Husserlian legacy.^76^

## Footnotes

1 Lukács 2008 [1949]; Scott 1992.

2 Cf. Apostolescu 2015, 24; Stoller 2009.

3 Thảo 1985, 39; Derrida 1973, 66-68; Scott 1992, 25; Foucault 1984, 2.

4 Mensch n.d.; Oksala 2014.

5 For examples of phenomenologists linking their work to other disciplines, see: Husserl 1977; Merleau-Ponty 1983; cf. Zahavi 2010. For examples of phenomenologists addressing social and political issues, see: Sartre 1974; Beauvoir 1996; Beauvoir 2010; Fanon 1970.

6 In a remarkably evocative end to his essay “Structure and Genesis in Husserl’s Phenomenology,” Welton (1977) suggests that “once perception is seen as a form of action, Husserlian phenomenology is on the way to an intellectual integration of perception and historical praxis” (67).

7 For examples discussing the critical phenomenological perspective, see: Weiss, Murphy, and Salamon 2020; Ferrari et al. 2018. For examples discussing interdisciplinary research collaboration, see: Burch 2021; Zahavi 2010; Gallagher 1997; Petitot et al. 1999.

8 Mason, Chowdhury, and Esner 2022, 31

9 For examples discussing these respective variants, see: Weiss, Murphy, and Salamon 2020; Zahavi 2021; Steinbock 2017, 1995; Varela 1996; Petitmengin, Remillieux, and Valenzuela-Moguillansky 2018.

10 Ahmed 2017, 15-16.

11 The two branches discussed here are illustrative of *contemporary* efforts to address these critical points, as pertaining to the conception of engaged phenomenology as an approach. It should be noted that these movements draw extensively from many predecessors whose work attempted to frame phenomenology as critique and praxis: see Fanon 1970, Sartre 1974, Merleau-Ponty 2014, Beauvoir 1996, and Thảo 1985, among others.

12 Davis 2020, 8.

13 Ibid; cf. Collins 2015.

14 hooks 1991, 2

15 Weiss, Murphy, and Salamon 2020.

16 Guenther 2020, 12-15.

17 Marx 1977 [1845], 158; Weiss, Murphy, and Salamon 2020, xiv.

18 Cf. Ferrari et al. 2018.

19 Oksala 2004, 20.

20 Cf. Rodemeyer 2006, 187.

21 Oksala 2004, 20.

22 Steinbock 2017, 90.

23 Ibid, 91.

24 Ibid.

25 For a critical-phenomenological analysis of the phenomenological method, including the status of the reduction and the epoché, see Davis (2020). As Merleau-Ponty (2014 [1945]) writes, rather than entirely separating the phenomenologist from the lifeworld, philosophical reflection “loosens the intentional threads that connect us to the world in order to make them appear” (Ixxvii).

26 For examples of phenomenological work heading in this direction, see discussion of Woods et al. 2014, Fernandez 2020, Stanier, Miglio, and Dolezal 2022, Miglio and Stanier 2022, and Vera-Gray 2020 in the section entitled “Two approaches to engaged phenomenology” below. While not necessarily amounting to collective critique or generation of meaning-complexes, it is also worth noting that critical and generative phenomenology are sometimes used as interpretative lenses by qualitative researchers: for instance, see Battalova et al. 2022 and Hvidt 2017.

27 Husserl 2001, 168.

28 Gallagher 2012, 308.

29 For example, see Merleau-Ponty 2014, 112; cf. Zahavi 2010.

30 Zahavi 2018; cf. Burch 2021.

31 For examples, see: Scholokhova, Bizzari, and Fuchs 2022; Køster and Fernandez 2021; Sass et al 2017; Høffding and Martiny 2015; Bevan 2014; Gallagher 2012; Petitmengin 2006; Parnas et al. 2005.

32 Zahavi 2018; Gallagher 2012.

33 Hurlbert and Schwitzgebel 2011; cf. Krueger, Bernini, and Wilkinson 2014; Alderson-Day and Fernyhough 2014.

34 Zahavi and Loidolt 2021, 27.

35 Petitmengin and Bitbol 2011; Hurlbert and Schwitzgebel 2011, 1; Gallagher and Francesconi 2012, 2

36 Petitmengin 2006, 248; Hulburt 2011, 66.

37 Hurlburt, Heavey, and Kelsey 2013.

38 Krueger, Bernini, and Wilkinson 2014, 9.

39 Levesque-Lopman 2000, 104.

40 For examples in social science literature, see: Iphofen and Tolich 2018; Riese 2019.

41 Cf. Dee-Price et al. 2021; Faulkner 2004.

42 Zahavi and Loidolt 2021, 27.

43 Woermann and Cilliers 2012, 448.

44 Heney and Poleykett 2022, 2.

45 Hall and Tandon 2017.

46 Spivak 1983, 90. Under such terms, marginalized people are often forced to only voice their own exclusion, echoing the voice of their interrogator, rather than speaking on and in their own terms. As Spivak puts it, the reflective orientation of a postcolonial intellectual would come from “seeking to learn to speak to (rather than listen to or speak for)” (ibid, 91)—not invoking others to speak for themselves, but rather by calling or appealing to them to respond.

47 Alcoff 1991; Haraway 1988. Lugones and Spelman (1983) describe the necessary ethical orientation towards research as an outsider in communities as follows: “This learning calls for circumspection, for questioning of yourselves and your roles in your own culture. It necessitates a striving to understand while in the comfortable position of not having an official calling card (as “scientific” observers of our communities have); it demands recognition that you do not have the authority of knowledge; it requires coming to the task without ready-made theories to frame our lives. This learning is then extremely hard because it requires openness (including openness to severe criticism of the white/Anglo world), sensitivity, concentration, self-questioning, circumspection. It should be clear that it does not consist in a passive immersion in our cultures, but in a striving to understand what it is that our voices are saying.” (581).

48 Mahmood 2011, 14-5.

49 Thomas et al. 2020; James n.d.; Williams et al. 2020a.

50 National Coordinating Centre for Public Engagement n.d.; Hinchliffe et al. 2018.

51 Durie, Wyatt, and Stuteley 2004.

52 Martin 2008; Rose 2014; Heney and Poleykett 2021, 9; Williams et al. 2020b.

53 Law 2004, 7.

54 Macmillan et al. 2012.

55 Steinbock 2017, 91.

56 Weiss, Murphy, and Salamon 2020, xiv.

57 Woods et al. 2014, S252.

58 Ibid. Here phenomenologists can perhaps follow critical historians (e.g. Goswami et al. 2014) and anthropologists (e.g. Harrison 1991) who have already developed methodologies pertaining to sensitive use of case studies from the context of their respective disciplinary approaches. Cf. Neale and Bishop 2012.

59 Stanier and Miglio 2021, 106.

60 Stanier, Miglio, and Dolezal 2022, 7.

61 Cf. Carel 2012.

62 Fernandez 2020, 21; cf. Callard et al. 2013; Miglio and Stanier 2022.

63 Rodemeyer 2006, 187.

64 Roth and Tobin 2004, 33.

65 Zahavi and Loidolt 2021, 27.

66 Vera-Gray 2020, 63.

67 Ibid; cf. Levesque-Lopman 2000, 104.

68 Woods et al. 2014, S252. See also work from the *Life of Breath* project, which also importantly foregrounds engaged and interdisciplinary methods: Malpass et al. 2019.

69 Beresford 2021.

70 Fricker 2007.

71 Weiss, Murphy, and Salamon 2020, xiv.

72 Heney and Poleykett 2021.

73 Attree et al. 2011.

74 Durie, Lundy, and Wyatt n.d.

75 This specific summary of engaged phenomenology was first published as the theme of the BSP Annual Conference in 2020, the Wolfe Mays Essay Prize in 2021, and the BSP Annual Conference again in 2022.

76 Ahmed 2006, 28.
